# Plasmid Dissemination in Multispecies Carbapenemase-Producing Enterobacterales Outbreaks Involving Clinical and Environmental Strains: A Narrative Review

**DOI:** 10.3390/microorganisms13040810

**Published:** 2025-04-02

**Authors:** Louis Alglave, Karine Faure, Catherine Mullié

**Affiliations:** 1 Centre d’appui pour la Prévention des Infections Associées aux Soins (CPias) Hauts-de-France, Home des Infirmiers, Avenue Oscar Lambret, 59037 Lille, Cedex, France; 2 EA7366, Translational Research Host-Pathogen Relation, Centre Hospitalier Universitaire de Lille, Université de Lille, 59000 Lille, France; 3 Infectious Disease Unit, Centre Hospitalier Universitaire de Lille, 59000 Lille, France

**Keywords:** carbapenemase, enterobacterales, outbreak, environment, horizontal gene transfer, plasmid, dissemination

## Abstract

Outbreaks involving carbapenemase-producing enterobacteria (CPE) have become a common occurrence in healthcare settings. While clonal dissemination is firmly established as a cause for these outbreaks, horizontal gene transfers (HGTs) between different species of Enterobacterales found in clinical and environmental isolates are less so. To gather evidence backing up this hypothesis, a review covering the 2013–2024 period was performed. HGTs between different species of clinical and environmental *Enterobacterales* were identified in thirteen papers, half of those published within the last three years. A combination of short- and long-read whole genome sequencing (WGS) was predominantly used to identify mobile genetic elements and plasmids. The more frequently reported carbapenemases were KPCs, followed by NDMs and IMPs. Predictably, broad-host-range plasmids were responsible for over 50% of HGTs, with the IncA/C group being in the lead. *Klebsiella pneumoniae* and *Enterobacter cloacae* complexes were the most frequent species identified in clinical samples, while *Citrobacter freundii* dominated environmental ones. Drains and pipework frequently constituted CPE reservoirs in protracted outbreaks, alternating epidemic outbursts with silent phases. Including WGS in a systematic environmental surveillance would help in swiftly identifying those CPE reservoirs and possibly help better control plasmid outbursts by allowing the implementation of adequate infection prevention and control measures.

## 1. Introduction

Since the first published reports on carbapenemase-producing bacteria (CPB) towards the end of the twentieth century, the health and economic burdens linked to these bacteria, especially carbapenemase-producing Enterobacterales (CPE), have been continually increasing [1,2,3]. The dissemination of CPB is such that they are nowadays not only the cause of numerous healthcare-associated infections (HAIs) but also of quite a few community-associated ones [4,5]. CPB represent a worrisome public health threat due to the reduced therapeutic alternatives available to treat the infections they cause. According to Ambler classification, the most prevalent carbapenemase enzymes are categorized in three groups within which five major families can be distinguished: the Ambler class A group with *Klebsiella pneumoniae* carbapenemases (KPCs); the Ambler class B group with metallo-ß-lactamases such as Imipenemases (IMPs), Verona Integron Metallo-β-lactamases (VIMs), and New-Delhi Metallo-β-lactamases (NDMs); and finally, the Ambler class D group with Oxacillinase-48 (OXA-48) and its variants [6]. In healthcare settings, CPE outbreaks have formerly been attributed to the dissemination of a given clone (as such termed clonal dissemination), due to the acquisition of specific virulence and/or persistence traits by this bacterial clone [7,8]. However, over the past decade, the rise in more comprehensive and affordable molecular biology techniques, such as short- and long-read whole genome sequencing, and of more powerful bioinformatic tools applied to bacterial outbreaks unraveled a potentially different landscape. Indeed, carbapenemase genes, the main drivers behind clinical carbapenem resistance, are generally encoded on mobile genetic elements (MGEs) such as plasmids, integrons, or transposons, thus enabling their horizontal transmission across species [9]. A recent survey estimated that around 44% of patients who acquired CPE met putative plasmid-mediated transmission criteria [10]. From an infection prevention and control (IPC) point of view, it appears crucial to identify whether the occurring outbreaks are mostly driven by clonal dissemination and/or horizontal gene transfer (HGT) to better adapt IPC responses. Indeed, HGTs are thought to occur both within an individual’s microbiomes, the intestinal microbiome being in the lead [11], and also within environmental biofilms [12,13]. Therefore, confinement and contact precautions around CPE-infected patients would not always be sufficient to check outbreaks involving HGTs. Moreover, it is sometimes even more difficult to identify an ongoing outbreak caused by HGTs, as mobile elements can be exchanged between various species within a given host and/or in the environment, possibly creating transmission reservoirs/sources that may persist over protracted periods of time. The main objective of this work was therefore to review the available evidence on outbreaks reported in healthcare settings for which such a transmission mode was pinpointed in multiple species, including strains found in the environment. Secondary objectives were to investigate whether a chronological pattern of interspecies transmission could be inferred from the selected studies and to identify whether a preferential species or environmental reservoir would stand out. These elements could indeed influence measures to be implemented to achieve the best possible IPC.

## 2. Materials and Methods

This narrative review was performed using the Pubmed database and citation searching. The search strategy aimed to target studies exploring outbreaks involving multiple CPE species in healthcare establishments, including molecular biology evidence and data from both clinical and environmental samples. Search terms were combined using Boolean operators and truncations to further refine the request and organized in seven sections. One section of the search equation focused on the orders, families, genera, and species of interest. The second targeted the resistances relevant to our investigation; the third emphasized the epidemic nature, followed by the anticipated modes of horizontal transmission, then the environmental aspect, and finally the nosocomial context. After reviewing the retrieved results with this first strategy, exclusion of terms related to wastewater and animals was added to filter titles. The final complete search strategy was written as follows: ((Escherichia[Title/Abstract]) OR (coli[Title/Abstract]) OR (Serratia[Title/Abstract]) OR (marcescens[Title/Abstract]) OR (Citrobacter[Title/Abstract]) OR (freundii[Title/Abstract]) OR (koseri[Title/Abstract]) OR (Klebsiella[Title/Abstract]) OR (pneumoniae[Title/Abstract]) OR (oxytoca[Title/Abstract]) OR (Enterobacter[Title/Abstract]) OR (cloacae[Title/Abstract]) OR (aerogenes[Title/Abstract]) OR (Morganella[Title/Abstract]) OR (morganii[Title/Abstract]) OR (Proteus[Title/Abstract]) OR (mirabilis[Title/Abstract]) OR (Enterobacteriaceae[Title/Abstract]) OR (Enterobacterales[Title/Abstract])) AND ((OXA-48*[Title/Abstract]) OR (NDM[Title/Abstract]) OR (VIM[Title/Abstract]) OR (IMP[Title/Abstract]) OR (KPC[Title/Abstract]) OR (lactamase*[Title/Abstract]) OR (carbapenem*[Title/Abstract])) AND ((transmission*[Title/Abstract]) OR (epidemic*[Title/abstract]) OR (outbreak*[Title/Abstract])) AND ((gene exchange*[Title/Abstract]) OR (plasmid*[Title/Abstract]) OR (conjugat*[Title/Abstract]) OR (Horizontal gene transfer*[Title/Abstract]) OR (biofilm*[Title/Abstract])) AND ((environment*[Title/Abstract]) OR (persisten*[Title/Abstract]) OR (surface*[Title/Abstract]) OR (water[Title/Abstract]) OR (soil[Title/Abstract]) OR (biofilm*[Title/Abstract])) AND ((hospital*[Title/Abstract]) OR (nosocomial*[Title/Abstract])) NOT ((waste*[Title]) OR (efflue*[Title]) OR (sewage*[Title]) OR (animal*[Title])). Original studies were eligible if published between 1 January 2013 and 31 December 2024. The search was last run against the database on 31 December 2024. The other inclusion criteria were as follows: any type of study design (randomized control trial, cohort study, case–control study, observational study, outbreak investigation); description of between-species/genera HGT of carbapenemase-encoding genes; study including both clinical and environmental isolates; and description of the methodology used for comparing plasmids/other mobile genetic elements. Studies with an English abstract were eligible when published in English or French. Publications were not eligible if they consisted of reviews, commentaries, and other opinion papers; did not include both environmental and clinical isolates; or described HGTs within a given species/complex or non-carbapenemase-encoding gene transfers.

After retrieval of results from the database and citation search, an independent screening of the papers based on the title and abstract was carried out by each reviewer to see if the selected articles could answer the purpose of this review. The reviewers then discussed discrepancies between their lists of papers to be included in the full-length article evaluation and settled on a common list of full-text articles to be evaluated. Similarly, the list of selected studies was jointly reappraised after reading full-length texts to agree on the final list of works to be included in this review.

The following pieces of information were sought and registered when available: geographical location of the work, time span of the study, ward/hospital setting, described species/genera, number of isolates per species/genus, isolate source (clinical/environmental), molecular biology techniques employed to assess both bacterial isolates and their mobile genetic elements, type of carbapenemase involved, and mobile genetic element involved.

## 3. Results

### 3.1. Main Characteristics of the Selected Studies

**Figure 1:**
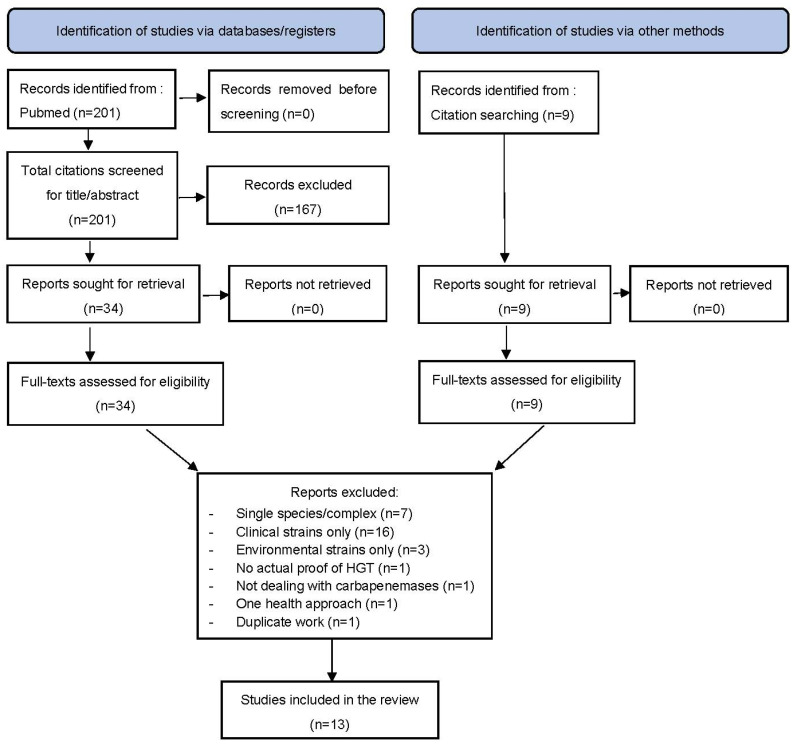
Multispecies carbapenemase-producing Enterobacterales outbreaks linked to plasmid dissemination between clinical and environmental strains. Flow chart based on recommendations from PRISMA 2020 [14].

Once the selection process was completed, 13 full-text papers were included in the analysis (Figure 1). Most of the studies consisted of outbreak investigations and/or of screening of banks of bacterial isolates (Table 1). Among the selected studies, European ones accounted for approximately half (46%) of the reports, followed by Northern American (31%), Australian (15%), and finally Asian (8%) ones (Table 1). The vast majority of reports originated from outbreak investigations (92%), and most outbreaks were circumscribed to a single hospital (69%). Wards in which outbreaks sprang were not systematically mentioned. However, it is noteworthy that, when this piece of information was made available (in 8 studies out of 13, 62%), 63% of the described outbreaks occurred in an Intensive Care Unit (ICU). The reported durations of outbreaks varied from 11 to 120 months (median: 34 months, interquartile range [17–94]). Over half of the included studies were published within the last three years of the investigated period (2013–2024).

### 3.2. Horizontal Transmission of Carbapenemase Genes

Table 2 summarizes the results from the 13 selected studies regarding carbapenemase types, sources, species, plasmids, and MGEs involved. As evidenced by the presence of a similar plasmid backbone and/or the same MGE in various species retrieved from clinical/environmental sources, HGTs were present in all 13 selected studies.

From the most to the least frequently encountered, carbapenemase families involved in HGTs among Enterobacterales were KPCs with four studies (31%), followed by NDMs and IMPs (three reports and 23% each), OXA-48-like carbapenemases (two reports, 15%), and finally VIM-1 (one study, 8%). Within the same family, two studies reported HGTs involving two different subclasses, namely IMP-1 and -11 [16] and KPC-2 and -3 [17]. Taking together patient and environmental isolates, the most frequently encountered species were those belonging to the *Klebsiella pneumoniae* complex and *Citrobacter freundii*, identified in 92% and 69% of the studies, respectively. The *Enterobacter cloacae* complex, *Escherichia coli*, and *Serratia marcescens* were also commonly found (in 62%, 62%, and 46% of the studies, respectively). When only environmental isolates are considered, the most frequently described species were *C. freundii* (five occurrences over the thirteen studies), followed by *K. pneumoniae* and *E. cloacae* complexes (four occurrences each). In a given species, similar isolates were found in clinical and environmental samples 17 times, including 4 times for *C. freundii* alone.

Classification of plasmids according to their incompatibility type was performed in all studies but one [21]. Plasmid profiling was either carried out by using S1-nuclease digestion followed by pulse field gel electrophoresis (PFGE), Southern blot and hybridization [15,18], PCR-based replicon-typing (PBRT) [15,18], and/or whole genome sequencing (WGS) [25] followed by in silico plasmid multi-locus sequence typing (pMLST) [16,24,26,27] or by in silico plasmid replicon typing (PRT) using PlasmidFinder [16,17,18,20,23,26,27] or Plasmid Profiler [19]. Discounting plasmids not assigned to a previously described incompatibility group (Types 1 and 2 [21] and RepA [17]), a total of 10 different incompatibility groups were identified. Multiple plasmid types were reported in six studies [16,17,18,19,21,23]. IncFII plasmids were the most frequently encountered (four occurrences), followed by IncA/C2, IncC, IncN, and IncM (two occurrences each). All other plasmid incompatibility types were only reported once.

Plasmid-mediated transmission of carbapenemase genes was predominantly described. Nevertheless, Phan et al. [16] reported the acquisition of a set of putative phage-associated elements, highlighting the involvement of phages in these genetic exchanges. Similarly, Gobeille-Paré et al. [19] demonstrated that the transfer of the OXA-204 mobile genetic elements was facilitated by a successful prophage, as well as three plasmids. Although the prophage was only observed in *Citrobacter freundii* isolates, the transfer of plasmids occurred between this species, *Klebsiella quasipneumoniae,* and *Escherichia coli*, underscoring the plasticity of these genetic elements and raising concerns about their potential spread.

## 4. Discussion

In this selection of works demonstrating HGTs between different species found both in environmental and clinical samples, KPC transmission was the most frequently reported, followed by NDM and IMP metallo-β-lactamases (MBLs). KPC carbapenemases are endemic in the United States of America and some southern European countries such as Greece and Italy [28]. Indeed, among the four reports of KPC HGTs included in this work, two originated from the United States while the two others were from Europe, albeit from northern European countries (Germany and Norway) rather than southern European ones. As OXA-48 and OXA-48-like carbapenemases are reported to have the highest prevalence in some European countries [28,29] and about half of the reports were from this geographical zone, these enzymes could have been expected to rank higher than the penultimate place in the occurrence frequencies. Instead, KPC and NDM carbapenemases were the most frequently reported in European studies. This might be due to a bias in publication, as greater attention would have been paid to outbreaks driven by less frequent carbapenemases in a given geographical area, and further investigations related to these outbreaks would have been more systematically carried out. Regarding MBLs, a recent literature review on the epidemiology of MBLs put forward that NDM isolates largely outnumber IMP and VIM ones (over 85% of the collected isolates vs. 8.9% and 5.6%, respectively) [6]. In our study, the occurrence frequency of reported HGTs was similar for NDM and IMP carbapenemases. This discrepancy might once more be linked to a publication bias associated with an over-representation of reports coming from Australia and Japan, which are known to have a high prevalence of IMPs among carbapenemases [6,28]. However, it cannot be ruled out that HGT frequencies among these various types of carbapenemases might not completely mirror the overall CPE epidemiology. In the case of NDMs, for example, bacteria carrying a *bla*_NDM_ genotype were shown to have significantly increased odds of clonal transmission compared with *bla*_KPC_ ones [10]. Finally, only one occurrence of VIM HGT was registered, coming from the United States of America.

As for plasmid distribution, even though techniques evolved over the period investigated in this review, most studies were able to classify identified plasmids according to incompatibility groups as described by Carattoli [30]. These incompatibility groups are in turn gathered in two clusters: broad-host-range and narrow-host-range plasmids [9]. Speaking of interspecies HGTs, incompatibility groups falling into the broad-host-range cluster would be expected to be more prevalent in the studies selected in this review. While 9 among the 17 described plasmids (53%) were actually broad-host-range and 2 (12%) displayed hybrid structures conferring broad-host-range distribution (IncU/IncX5 and IncFII/FIB/A/C2), 6 plasmids (35%) had, however, a strictly narrow-host-range. Within this subset, four belonged to the IncFII group. IncFII plasmids often harbored insertion sequences such as IS*Kpn26* and IS*Ehe3*, which facilitate gene mobilization and recombination events [16]. KPC dissemination has been predominantly linked with IncF, IncN, and IncX plasmids, while NDM carbapenemase genes have mostly been described in IncF, IncX, and IncA/C plasmids [9]. These patterns were respected in studies selected in this review, with the exception of one NDM gene harbored on an IncN plasmid. IMP-carrying plasmids have previously been mainly allocated to IncHI and IncP groups [9]. The studies collected in this review reported a single occurrence of IMP transmission through an IncHI plasmid, while the other four IMP-carrying plasmids belonged to broad-host-range groups C, L/M, M1, and M2. Similarly, the two carbapenemases of the OXA-48 family were not found to be transmitted through IncX or IncL/M plasmids as reported earlier [9] but on an A/C2 and a hybrid FII/FI/A/C2 one. These discrepancies might be linked to the main objective of this review (i.e., to describe outbreaks with interspecies dissemination of carbapenemase genes through plasmids) as IncHI is a narrow-host-range group. Another explanation could be an actual switch in plasmids predominantly disseminating IMP genes over time. Indeed, broad-host-range plasmids IncA/C and L/M were more frequently encountered while drawing up this review. Moreover, most of the works included here postdate the ones over which the review by Kapotsa et al. [9] was built. Another point to take under consideration is the complexity of genetic adaptations that is not entirely reflected by the attribution of a given plasmid to an incompatibility group. To better describe evolutionary trajectories underlying such outbreaks, a systematic phylogenetic analysis of both the whole bacterial genome and plasmids would be of great worth. Some studies have already endeavored to carry out so [16,19,20,22,23,27]. It has enabled the identification of recombination events and mosaic/hybrid plasmids [19,23]. It can also bring light to the role of bacteriophages [16,19] and other MGEs in carbapenem-resistance dissemination.

The secondary objectives of this work were (i) to establish whether a “typical” chronological pattern of interspecies transmission could be inferred from the selected studies and (ii) whether some species/environmental locations were more prone to act as reservoirs for carbapenemase genes. While some studies chronologically followed patients and found evidence of colonization or infection with different bacterial species over time [19,21,24,25], most reviewed studies did not provide a clear temporal order in which patients/the environment became colonized/infected with CPE. It therefore seemed inadequate to try and draw conclusions on such a limited sample of observations. As for species distributions in clinical and environmental isolates, it has to be first underscored that the total number of environmental isolates was much lower than the clinical ones, largely due to the fact that environmental sampling was not systematically performed in most work. When clinical isolates are concerned, *K. pneumoniae*, *E. coli*, *C. freundii*, and *E. cloacae* were the most frequently reported species/complexes. However, a substantial diversity beyond these classic carbapenemase-producing (CP) species was observed, with several studies also reporting on clinical *S. marcescens*, *Klebsiella oxytoca*, and *Klebsiella michiganensis* isolates. Seldom isolated CP species included *Morganella morganii* and *Citrobacter amalonaticus*, but also species usually mostly isolated from environmental and/or food samples: *Leclercia adecarboxylata*, *Raoultella ornithinolytica*, or *C. portucalensis* [31,32,33]. As for environmental isolates, it is noteworthy that *C. freundii* was the leading species identified in those isolates and that it contributed to a fourth of the strains found in aggregated clinical and environmental samples. The possible pivotal role of this peculiar species in HGTs and protracted outbreaks has recently been highlighted in papers reporting its prolonged persistence in healthcare environments and in water [20,34,35]. In environmental samples, *K. pneumoniae* and *E. cloacae* complexes were the second and third most frequent species, mirroring their predominance in clinical isolates. A couple of environmental isolates were also witnessed for *S. marcescens*, *E. coli*, and *Klebsiella michiganensis*, supporting the idea of a wider spectrum of *Enterobacterales* playing a role in environmental persistence and interspecies gene transfers.

The most frequent locations in which CPB were found in the environment were moist ones, including sinks and sink drains [15,17,21,22,23,24,25,26]. These locations have already been pointed out as a potential reservoir for CPE, especially in ICUs [36,37]. This observation makes sense, as a good number of the outbreaks included in this review occurred in ICUs. Other water-associated reservoirs were also examined, such as rinse water [18], sink siphon water [27], bathrooms, showers, sluices, and taps [19,20], or hygiene tank traps [19]. Sink drains and plumbing represent a difficult-to-treat reservoir for ongoing gene exchange [37]. IPC measures therefore often target the wet environment and plumbing, including replacement of sink drains and pipework, chlorine/hydrogen peroxide/acetic acid/enzymatic/vapor/ozone decontamination of sink drains, addition of heating–vibrating devices on sink traps, and waterless patient care, among others [15,24,25,26,38,39]. Successful control of outbreaks, including such environmental reservoirs, often involves the need for combined measures that have to be upheld over long periods of time [24,39]. Nevertheless, success of multimodal IPC measures on sink drains and pipework is not a foregone conclusion, with recontaminations of sink drains regularly being witnessed [15,20,21,24,26].

As it has already been pointed out before [36], due to the retrospective nature of most outbreak investigations, the lack of a systematic timeline for the retrieval of environmental and clinical isolates does not help in answering the key question of whether environmental contamination is first the source of patient colonization or whether patients first contaminate their surroundings. However, once this first occurrence takes place, the most plausible scenario is a bidirectional process, in which contamination cycles between patients and their environment recurrently happen [17,37]. Most studies reporting protracted outbreaks related prolonged periods of time over which no new clinical contamination/transmission could be detected [15,17,19,20,21,22,23]. Clinically silent environmental niches can generate epidemic bursts, sometimes weeks or even months apart, and sometimes with a different species than the original one. From an IPC point of view, predicting and controlling such resurgences is challenging. Even more so when the reemerging CP species is different from that isolated in the former bout of the outbreak, rendering the control of so-called plasmid(-borne) outbreaks [15,21] quite complicated. The advent of WGS techniques has given the means to better decipher the transmission routes of MGEs between species, enabling a better identification of those plasmid outbreaks. Nowadays, the main drawback of these techniques is their cost and availability. However, due to their unmatched accuracy, replacing them with equally efficient but less costly and more readily available methods is difficult. Nevertheless, some works have already been published focusing on the cost-effectiveness of WGS patient-screening programs for IPC with promising results [40,41]. Further studies are still needed to draw up a solid scheme for systematic environmental screening coupled with WGS techniques (possibly focusing on moist environmental niches and/or specific units such as ICUs) and assess its cost-effectiveness and place in IPC strategies.

## 5. Conclusions

In recent years, the generalization of short- and long-read WGS has enabled an increase in reports demonstrating the transfer of carbapenemase genes between both clinical and environmental isolates belonging to different species of Enterobacterales during nosocomial outbreaks. Swiftly identifying those plasmid-borne outbreaks is of great worth for the implementation of adequate IPC measures. Indeed, while it appears highly difficult to impede HGTs occurring within an individual, raising awareness of HGTs occurring in environmental niches is of paramount importance. Future works assessing systematic and standardized environmental surveillance schemes, coupled with an appropriate use of WGS, would help assess whether such measures can rapidly identify possibly hidden threats and give better odds to contain such plasmid-borne CPE outbreaks.

## Figures and Tables

**Table 1:** Main characteristics of the studies included in this review.

Reference	Continental Location (Country)	Isolates Retrieved from	Setting	Duration in Months (Period Dates)
Tofteland et al., 2013 [15]	Europe (Norway)	1 OI R and P CSP 2 ESC	1 ICU * (outbreak), 1 hospital (environmental screening) 3 hospitals (clinical screening)	11 (05/2010–04/2011)
Phan et al., 2018 [16]	Europe (Romania)	2 OIs 1 ESC	1 NICU, 1 neonatal ward 2 hospitals	60 (2010–2015)
Mathers et al., 2019 [17]	America (United States)	CSP 1 ESC	1 hospital	117 (08/2007–05/2017)
Weber et al., 2019 [18]	Europe (Germany)	1 OI CSP ESC	7 wards 1 hospital	27 (05/2015–09/2017)
Gobeille-Paré et al., 2020 [19]	America (Canada)	1 OI CSP ESC	ICU, Nephrology unit 4 hospitals	34 (01/2016–10/2018)
Kizny-Gordon et al., 2020 [20]	Australia (Australia)	1 OI ESCs	1 burn unit 1 hospital	120 (2006–2015)
Brehony et al., 2021 [21]	Europe (Ireland)	1 OI ESCs	1 (confined) ward 1 hospital	18 (07/2018–12/2019)
De Man et al., 2021 [22]	America (United States)	1 OI ESC	1 ICU 1 hospital	10 (08/2015–05/2016)
Macesic et al., 2023 [23]	Australia (Australia)	1 OI ESC	1 hospital	96 (2002–2020)
Anantharajah et al., 2024 [24]	Europe (Belgium)	1 OI CSP ESCs	1 ICU 1 hospital	48 (01/2018–12/2022)
Rankin et al., 2024 [25]	America (United States)	2 OIs CSP ESCs	1 hospital	17 (07/2017–12/2018)
Tsukada et al., 2024 [26]	Asia (Japan)	1 OI Post-OI P CSP ESC	1 pediatric ward 1 hospital	17 (06/2016–10/2017)
Yao et al., 2024 [27]	Europe (Germany)	OIs SP ESC	61 hospitals	94 (2013–2019)

* Abbreviations used in this table: CSP: clinical screening program; ESC: environment screening campaign; NICU: Neonatal Intensive Care Unit; ICU: Intensive Care Unit; OI: outbreak investigation; P: prospective; R: retrospective; SP: surveillance program.

**Table 2:** Evidence supporting horizontal gene transfer (HGT) between clinical and environmental Enterobacterales.

Reference	Carbapenemase	Sources	Species	Plasmid		Involved Mobile Genetic Elements
				Length (kb)	Grouping	
Tofteland et al., 2013 [15]	KPC-2	P + E *	*K. pneumoniae*	97	IncFII	ND
		P + E	*E. asburiae*			
Phan et al., 2018 [16]	NDM-1	P	*S. marcescens*	ND	IncFII	IS*Kpn26*, IS*Ehe3*
		P	*E. cloacae*			
		P	*K. pneumoniae*			
		E	*S. marcescens*		ND	
Mathers et al., 2019 [17]	KPC-3	P	*K. quasipneumoniae*	447	RepA	Tn*4401b-1*
	KPC-2 + KPC-3	E	*K.* *quasipneumoniae*	447 53	IncU/X5 RepA	
	KPC-2	E	*K. quasipneumoniae*	441 69	IncX5 RepA	
	KPC-2	P	*S. marcescens*	69	IncU/X5	
Weber et al., 2019 [18]	NDM-1	P + E	*E. coli*	44.5	IncN	Tn*125*, IS*26*
		P	*M. morganii*			
		P	*E. coli*	115.3	IncA/C2	
		P	*K. pneumoniae*	155.2		
		P	*C. freundii*	176.5		
Gobeille-Paré et al., 2020 [19]	OXA-204	P + E	*C. freundii*	256–282	IncFII/FIB/A/C2	ND
		P	*E. coli*			
		P	*K. quasipneumoniae*	187	IncA/C2	
Kizny-Gordon et al., 2020 [20]	IMP-4	P	*E. cloacae*	88 173	IncM2	*blaIMP-4-qacG2-aacA4-catB3* cassette
		E	*E. cloacae*	86		
		P	*C. freundii*	176		
		E	*C. freundii*	87 176		
		P	*K. pneumoniae*	87 209		
		P	*K. oxytoca*	59		
		E	*S. marcescens*	87		
		E	*L. adecarboxylata*	128 82		
		E	*Enterobacter spp.*	96		
		P	*E. coli*	ND		IS*116*, IS*110*, IS*902*
		P	*S. marcescens*			
Brehony et al., 2021 [21]	OXA-48	P	*E. cloacae complex*	ND	Types 1 and 2	ND
		P	*E. coli*			
		E	*K. michiganensis*		Type 1	
		P	*K. michiganensis*		Type 2	
		P	*K. oxytoca*			
		P	*C. freundii*			
		P	*S. marcescens*			
De Man et al., 2021 [22]	VIM-1	P	*K. pneumoniae*	160 164	IncA/C2	In*1209*
		P	*E. hormaechei*			
		P	*E. coli*	160		
		P	*R. ornithinolytica*			
		E	*C. amalonaticus*			
Macesic et al., 2023 [23]	IMP-4	P + E	*E. hormaechei*	ND	IncC IncHI2A IncL/M	Class 1 integron *blaIMP-4-qacG-aacA4-catB3-qacE-sul1*
		P	*E. cloacae*		IncC IncL/M	
		P	*E. coli*			
		P	*K. pneumoniae*			
		P	*K. michiganensis*			
		P	*C. freundii*			
		P	*K. oxytoca*		IncC IncHI2A	
		P + E	*S. marcescens*		IncC	
Anantharajah et al., 2024 [24]	NDM-1	P + E	*E. cloacae*	140	IncC	Class 1 integron
		P + E	*C. freundii*			
		P	*E. coli*			
		P + E	*K. oxytoca*			
		P	*P. mirabilis*			
		P	*K. pneumoniae*			
Rankin et al., 2024 [25]	KPC-3	P + E	*C. freundii*	ND	IncFII	ND
		E	*E. cloacae complex*			
		P	*S. marcescens*			
Tsukada et al., 2024 [26]	IMP-1	P + E	*K. pneumoniae complex*	ND	IncM1	Class 1 integron
		P	*C. freundii*			
	IMP-11	P	*E. cloacae complex*			
Yao et al., 2024 [27]	KPC-2	P	*K. pneumoniae*	ND	IncN	NTE*_KPC_*-Y cassette, IS*26*
		P + E	*E. coli*	43-kb backbone		
		P + E	*C. freundii*			
		P + E	*E. xiangfangensis*			
		P + E	*K. michiganensis*			
		P + E	*C. portucalensis*			
		P + E	*K. aerogenes*			
		P	*C. koseri*			

* Abbreviations used in this table: E: environment; In: integron; Inc: incompatibility group; IS: insertion sequence; ND: not determined; P: patient; Tn: transposon.

## Data Availability

The original contributions presented in this study are included in this article. Further inquiries can be directed to the corresponding author.
